# Interactions of the Kv1.1 Channel with Peptide Pore Blockers: A Fluorescent Analysis on Mammalian Cells

**DOI:** 10.3390/membranes13070645

**Published:** 2023-07-04

**Authors:** Nikita A. Orlov, Elena V. Kryukova, Anastasia V. Efremenko, Sergey A. Yakimov, Victoria A. Toporova, Mikhail P. Kirpichnikov, Oksana V. Nekrasova, Alexey V. Feofanov

**Affiliations:** 1 Shemyakin-Ovchinnikov Institute of Bioorganic Chemistry, Russian Academy of Sciences, 117997 Moscow, Russia; n.orlov858@yandex.ru (N.A.O.); evkr@mail.ru (E.V.K.); aefr@mail.ru (A.V.E.); sa-yakimov@yandex.ru (S.A.Y.); toporova.viktorija@gmail.com (V.A.T.); kirpichnikov@inbox.ru (M.P.K.); avfeofanov@yandex.ru (A.V.F.); 2 Faculty of Biology, Lomonosov Moscow State University, 119234 Moscow, Russia

**Keywords:** Kv1.1 channel, pore blocker, fluorescence, confocal, patch clamp, affinity, hongotoxin, competitive binding

## Abstract

The voltage-gated potassium channel Kv1.1, which is abundant in the CNS and peripheral nervous system, controls neuronal excitability and neuromuscular transmission and mediates a number of physiological functions in non-excitable cells. The development of some diseases is accompanied by changes in the expression level and/or activity of the channels in particular types of cells. To meet the requirements of studies related to the expression and localization of the Kv1.1 channels, we report on the subnanomolar affinity of hongotoxin 1 N-terminally labeled with Atto 488 fluorophore (A-HgTx) for the Kv1.1 channel and its applicability for fluorescent imaging of the channel in living cells. Taking into consideration the pharmacological potential of the Kv1.1 channel, a fluorescence-based analytical system was developed for the study of peptide ligands that block the ion conductivity of Kv1.1 and are potentially able to correct abnormal activity of the channel. The system is based on analysis of the competitive binding of the studied compounds and A-HgTx to the mKate2-tagged human Kv1.1 (S369T) channel, expressed in the plasma membrane of Neuro2a cells. The system was validated by measuring the affinities of the known Kv1.1-channel peptide blockers, such as agitoxin 2, kaliotoxin 1, hongotoxin 1, and margatoxin. Peptide pore blocker Ce1, from the venom of the scorpion *Centruroides elegans*, was shown to possess a nanomolar affinity for the Kv1.1 channel. It is reported that interactions of the Kv1.1 channel with the studied peptide blockers are not affected by the transition of the channel from the closed to open state. The conclusion is made that the structural rearrangements accompanying the channel transition into the open state do not change the conformation of the P-loop (including the selectivity filter) involved in the formation of the binding site of the peptide pore blockers.

## 1. Introduction

The Kv1.1 channel, a member of a large group of voltage-gated potassium channels, is distributed mainly in the brain (hippocampus, cerebellum, neocortex) and peripheral nervous system and is considered a critical regulator of neuronal excitability [1]. Kv1.1 channels, being localized in the initial axon segments and in the juxtaparanodal region of axons, stabilize axonal conduction [2] and also regulate neurotransmitter release in nerve terminals [3]. Recent studies revealed the role of the Kv1.1 channel in the secretion of insulin by pancreatic β-cells [4] and in controlling the smooth muscle tone in arteries [5]. Expression of the Kv1.1 channel was found in normal helper T lymphocytes, where they are involved in the regulation of the production and secretion of TNFα cytokine [6].

Kv1.1 is a low-threshold channel, which is rapidly activated by small changes of transmembrane potential and generates a slowly inactivating current. The functional activity of the Kv1.1 channel is modulated by various mechanisms, including association with auxiliary Kvβ1 subunits or α-subunits of the Kv1.4 channel, which confer functional plasticity to Kv1.1 in controlling the action potential threshold, its duration, and firing frequency [1,7]. The one-transmembrane regulatory protein KCNE4 was concluded to drastically inhibit Kv1.1 channel currents, presumably by affecting the channel gating [8]. Intracellular trafficking, and the mechanisms that regulate cell surface expression of Kv1.1 channels, also exert their influence on neuronal signaling. Homomeric Kv1.1 channels are mainly localized in the endoplasmic reticulum (ER), and the ER retention signal for the Kv1.1 channel was mapped in the P-loop of its pore domain [9,10]. Heteromerization of the Kv1.1 channel with other members of the Kv1 channel family increases its membrane expression [9,10].

Further study of the Kv1.1 channel, in terms of its tissue distribution and localization, association with particular types of cells, as well as functional variations in the membrane expression of the channel, can be assisted with the development of bright high-affinity fluorescent ligands of this channel. In the present paper, we characterize hongotoxin 1 N-terminally labeled with Atto 488 fluorophore (A-HgTx) as such a ligand and demonstrate its applicability for the fluorescent imaging of Kv1.1 in living cells.

Involvement of the Kv1.1 channel in various physiological processes predefines its pharmacological significance. Inherited mutations in the *KCNA1* gene encoding the Kv1.1 channel cause a number of disorders, which are linked with increased excitability of neurons. Among them are episodic ataxia type I, which is characterized by motor dysfunction and epilepsy [1]. A decrease in the expression of the Kv1.1 channel can induce neuronal hyperexcitability and impair inhibitory synaptic transmission, causing epileptic seizures as demonstrated with the *KCNA1* knock-out mouse model (*KCNA1*^−/−^) [11]. Inhibition of the Kv1.1 channel in normal helper T lymphocytes results in the production and secretion of a pro-inflammatory cytokine TNFα that may cause or exacerbate autoimmune/inflammatory diseases but have a therapeutic effect in the case of cancer or bacterial infections [6]. Another example of the pharmacological importance of the Kv1.1 channel is its ectopic expression in demyelinated axons, which is the cause of abnormal neuronal conductivity [12,13]. Blocking of the Kv1.1 channel improved impaired axonal function, thus demonstrating the therapeutic potential of this approach to alleviate symptoms in multiple sclerosis [14].

The cited data indicate that modulation, in particular, inhibition of Kv1.1 activity, may be useful in the therapy for certain diseases. Inhibition of Kv1.1 can be achieved, for example, with high-affinity peptide pore blockers, which are found in the venoms of snakes and scorpions and considered as a basis for drug development. Along with 57–60-aa dendrotoxins from snake venom [15], many shorter peptides (37–39 aa) from the α-KTx family of scorpion toxins are blockers of the Kv1.1 channel [16]. However, neither of the α-KTx toxins was shown to be a selective Kv1.1 channel blocker since these peptides usually share specificity for the highly homologous channels Kv1.1, Kv1.2, Kv1.3 and (less often) Kv1.6. The development of Kv1.1-selective peptide blockers is required, along with the development of Kv1.3-selective blockers. In the latter case, inhibition of the Kv1.3 channel, a drug target in autoimmune diseases [17], should be carried out without unwanted Kv1.1-related production of pro-inflammatory cytokine TNFα [6].

Besides the potential clinical applications, peptide blockers are widely used in ion channel research; thus, the search for new such peptides is expanding and requires various convenient methods for their study. In addition to classical electrophysiological methods [18], radioligand analysis [19,20], and Rb^+^ (or ^86^Rb) efflux assay [21,22], a new technique was recently proposed, which is based on the confocal microscopy analysis of competition between the studied peptide and fluorescent ligand for the binding to the fluorescent ion channel on the plasma membrane of cells. It was realized for the Kv1.3 channel [23].

In the present study, we report on the analogous fluorescence-based analytical system, which was developed to study the outer pore ligands of the Kv1.1 channel. The system includes A-HgTx and the Kv1.1 channel fused with the mKate2 fluorescent protein, which is presented in the plasma membrane of neuroblastoma Neuro2a cells. The high level of presentation of the human fluorescent Kv1.1 channel in the cellular membrane was achieved due to the S369T mutation. The functionality of this channel, and its ability to bind peptide pore blockers, was confirmed by the patch-clamp technique. The affinities of several peptide pore blockers for the Kv1.1 channel were measured with the developed analytical system. An experimental approach was worked out to compare the affinities of the peptide blockers for the Kv1.1 channel in the open and closed states.

## 2. Materials and Methods

### 2.1. Reagents

HgTx1 labeled with Atto488 at the N-terminus (A-HgTx, purity 98%) was from Smartox Biotechnology (Saint Egrève, France). Its concentration was determined with spectrophotometry using a molar extinction coefficient of 90,000 M^−1^cm^−1^ at 500 nm. GenJector-U Transfection Reagent was from Molecta (Moscow, Russia). LysoTracker Green (LTG), NBD-labeled C6-Ceramide (NCer), ER Tracker Green (ERTG), and transferrin labeled with Alexa Fluor 488 (TR488) were from ThermoFisher Scientific (Waltham, MA, USA). Bovine serum albumin (BSA) and rhodamine 123 (Rh123) were from Merck (Darmstadt, Germany). Oligonucleotide primers (Table 1) were synthesized by Evrogen (Moscow, Russia). Tobacco etch virus (TEV) protease was produced and purified as described elsewhere [24] using *E. coli* BL21(DE3) CodonPlus-RIL cells containing pRK793 plasmid coding for His6-TEV(S219V)-Arg5 protease (AddGene, Inc., Cambridge, MA, USA).

### 2.2. Design of Expression Plasmids Encoding Wild Type and Mutated Kv1.1 N-Terminally Tagged with a Fluorescent Protein

*KCNA1* gene of the human Kv1.1 channel (accession number NP_000208.2) was received from Dr. A. Vassilevski. The *KCNA1* gene was amplified in PCR using Kcna1-f1 and Kcna1-r1 (Table 1), and the obtained DNA was cloned into BglII/HindIII sites of expression vector pmKate2-C (Evrogen, Russia) in order to get the pmKate2-KCNA1 plasmid, which encoded Kv1.1 having mKate2 at the N-terminus (K-Kv1.1wt).

A single nucleotide substitution (TCC to ACC) was introduced into *KCNA1* to produce the pmKate2-KCNA1m1 plasmid, which encoded the hKv1.1S369T mutant with mKate2 at the N-terminus (K-Kv1.1). For this, site-directed mutagenesis by overlap extension was used with two mutagenic primers, Kcna1m1-f1 and Kcna1m1-r1, which have a 22-bp overlap with each other at their 5′ ends (Table 1). In the first round of polymerase chain reaction, two pairs of oligonucleotide primers (Kcna1-f1 and Kcna1m1-r1; Kcna1m1-f1 and Kcna1-r1) were used separately to obtain two DNA fragments (1120 and 390 bp in length, respectively) from the pmKate2-KCNA1 plasmid used as a template. Then, these fragments were combined and amplified using terminal primers to obtain the whole mutated gene. The gene was cloned in the pmKate2-C vector at BglII/HindIII sites.

To construct the pTagCFP-KCNA1m1 plasmid, the gene encoding the TagCFP fluorescent protein was amplified from the pTagCFP-C plasmid (Evrogen, Russia) using primers Fl-f1 and Cfp-r1 (Table 1) and then cloned into NheI/BglII sites of pmKate2-KCNA1m1. The resulting pTagCFP-KCNA1m1 plasmid-encoded C-Kv1.1, the hKv1.1S369T mutant N-terminally tagged with TagCFP through the GGGGSG spacer. The spacer was the same as in K-Kv1.1. The sequences of wild-type and mutated *KCNA1* genes, as well the *TagCFP* gene sequence, were confirmed in the created plasmids (pmKate2-KCNA1, pmKate2-KCNA1m1, and pTagCFP-KCNA1m1, respectively) by sequencing both strands according to Sanger, which was performed by Evrogen (Russia).

### 2.3. Recombinant Peptide Toxins and GFP-Tagged AgTx2

Margatoxin (MgTx), kaliotoxin 1 (KTx1), charybdotoxin (ChTx), agitoxin 2 (AgTx2), and hongotoxin 1 (HgTx1) were expressed and purified as described previously [25]. Using the same bioengineering strategy, a recombinant toxin Ce1 from the scorpion *Centruroides elegans* [26] was produced. For this, the gene encoding Ce1 fused with the TEV protease cleavage site (CS_TEV_) at its N-terminus (ENLYFQTVINVKCTSPKQCLKPCKDLYGPHAGAKCMNGKCKCYNN, the sequence of CS_TEV_ is underlined; the residue in the P1’ position of the cleavage site is occupied by the N-terminal residue of Ce1 peptide) was synthesized by PCR using two forward oligonucleotide primers Ce1-f1 and Ce1-f2, and two reverse primers Ce1-r1 and Ce1-r2 (Table 1). The DNA fragment was cloned into KpnI and HindIII restriction sites of the pET-23d-MalE-CS_TEV_-AgTx2 plasmid [25] to obtain pET-23d-MalE-CS_TEV_-Ce1, where *MalE* is a gene encoding maltose binding protein (MBP). The fusion protein MBP-CS_TEV_-Ce1, in which a 45 aa linker sequence between MBP and CS_TEV_-Ce1 carried the His6 tag, was expressed in the *E. coli* strain Rosetta-gami(DE3)pLysS (Novagen Merck KGaA, Darmstadt, Germany) and after purification on a Ni Sepharose Fast Flow column (GE Healthcare, Chicago, IL, USA) was cleaved with the TEV protease. The products of the hydrolysis were fractionated by reverse-phase (RP) HPLC using a Discovery Bio Wide Pore C18 column (15 cm × 4.6 mm, 5 μm, MilliporeSigma Supelco, Burlington, MA, USA) in a gradient of 0–50% acetonitrile in the presence of 0.1% trifluoroacetic acid. Purified Ce1 peptide was lyophilized, dissolved in water, and stored at −25 °C. The molecular weight of Ce1 measured by MALDI-TOF mass spectrometry was 4253 Da, which corresponds to the calculated molecular weight value of a peptide with three disulfide bridges. The yield of Ce1 was 6.5 mg from 1 L of culture.

UV spectrophotometry and molar extinction coefficients 54,600, 54,700, 49,300, 49,200, 282,000, and 55,800 M^−1^ cm^−1^ (λ = 214 nm) were used to measure concentrations of HgTx1, MgTx, KTx1, AgTx2, ChTx, and Ce1, respectively. The calculation of these coefficients was performed as described earlier [27]. Measurements were carried out in water mixed with trifluoroacetic acid (0.1%) and acetonitrile (20%).

### 2.4. Cells and Experiments with Cells

Mouse neuroblastoma Neuro2a cells and HEK293 cells (from the Russian collection of cell cultures, the Institute of Cytology RAS, Saint Petersburg, Russia) were grown in the Dulbecco’s modified Eagle’s medium (DMEM/F12) containing 2 mM L-glutamine (Paneco, Moscow, Russia) and 5% fetal bovine serum (FBS, HyClone, Logan, UT, USA) (hereinafter referred as a complete medium). The passage of cells was performed twice a week.

Cells were grown on round glass coverslips pre-coated with poly-D-lysine in the 24-well plates ((3 ± 1) × 10^4^ or (6 ± 1) × 10^4^ cells per well of Neuro2a or HEK293 cells, respectively). Transfection of cells with pmKate2-KCNA1, pmKate2-KCNA1m1, or pTagCFP-KCNA1m1 plasmid was performed with GenJector-U reagent according to the manufacturer’s protocol at 30–40% cell confluence. The experiments were conducted 48 h after transfection.

Cellular organelles were stained in a complete medium, as described previously [23].

To analyze the binding of A-HgTx, A-HgTx (0.25–4 nM) was added to cells in a complete medium for 60 min. To analyze competitive binding, A-HgTx (2 nM), together with the selected peptide (0.05–160 nM), was added to cells in a complete medium for 60 min.

To compare ligand binding with closed and open channels, ligands were added to cells (60 min, 37 °C) in a buffer (20 mM HEPES pH 7.4, 0.9 mM CaCl_2_, 0.5 mM MgCl_2_, 0.1% BSA) supplemented with 4 mM KCl and 150 mM NaCl (Na^+^ buffer) or with 150 mM KCl (K^+^ buffer).

### 2.5. Electrophysiology Measurements

Membrane ion currents through the K-Kv1.1 channel were recorded at room temperature by the patch-clamp technique using the standard whole-cell configuration as described earlier [23].

Briefly, Neuro2a cells attached to coverslips (10 mm in diameter) pre-coated with poly-D-lysine were immersed in a buffer (140 mM NaCl, 2.8 mM KCl, 2 mM MgCl_2_, 2 mM CaCl_2_, 10 mM glucose, 10 mM HEPES, pH 7.4) and perfused continuously. Micropipettes with a tip resistance of 6–8 MΩ were filled with a pipette solution containing 140 mM KCl, 6 mM CaCl_2_, 2 mM MgCl_2_, 2 mM MgATP, 0.4 mM NaGTP, 10 mM HEPES, 20 mM BAPTA/KOH (pH 7.3). The patches were routinely held at a holding potential of −40 mV. The potential was changed from −70 to +70 mV (20 mV step) using 200 ms pulses, which were applied incrementally with an interval of 20 s. Finally, the potential returned to the holding potential.

Cells were measured 24–48 h after transfection. The cells were selected by K-Kv1.1 fluorescence. The cells were analyzed if their input resistance was more than 350 MΩ. The currents were recorded using an EPC-10 amplifier (HEKA Elektronik, Stuttgart, Germany).

The inhibitory activity of the toxins was assessed by applying HgTx1 (2 nM) or fluorescently labeled A-HgTx to the cell in an increasing concentration (2–15 nM). The toxins were dissolved in a complete medium.

The measured concentration dependence of current inhibition through the K-Kv1.1 channel by A-HgTx was analyzed with the following equation:

*I_rel_*(*L*) = 100/(1 + (*IC*_50_/*L*)*^h^*) (1)

where *I_rel_* is a decrease in current (%), *L* is the concentration of A-HgTx, *IC*_50_ is the concentration of A-HgTx that induces a 50% decrease in current, and *h* is the Hill coefficient.

### 2.6. Confocal Microscopy

The studies were performed using a laser scanning confocal microscope SP2 (Leica Microsystems GmbH, Wetzlar, Germany) with a 63× water-immersion objective (HCX PL APO, NA = 1.2). The resolution in the axial and lateral directions was 0.6 and 0.2 µm.

The fluorescence of the CFP-Kv1.1 channel was excited at 458 nm and recorded in the 470–600 nm range.

The fluorescence of the K-Kv1.1 and K-Kv1.1wt channels was excited at 561 nm and detected in the 650–700 nm region. A-HgTx fluorescence was excited at 488 nm and detected in the 495–535 nm region. Sequential scanning was used to measure the cellular distributions of A-HgTx and mKate2 fluorescence in order to avoid interference of mKate2 fluorescence with cellular autofluorescence excited at 488 nm.

Sequential scanning was also used to measure confocal images of the K-Kv1.1 channel and fluorescent probes of cellular organelles. The fluorescent probes were excited at 488 nm and detected in the 498–550 nm region.

### 2.7. Quantitative Analysis of Pore Blocker Interactions with K-Kv1.1

Quantitative analysis of the interaction of A-HgTx with K-Kv1.1 was carried out in accordance with the procedure developed previously for A-HgTx and the fluorescently labeled Kv1.3 channel [23]. Briefly, the regions of interest with K-Kv1.1 on the plasma membrane were selected in the confocal images and used to create binary (0;1) masks that described the membranous distribution of K-Kv1.1. The confocal images of K-Kv1.1 and A-HgTx were multiplied by the corresponding mask, and the average fluorescence intensities of A-HgTx (*I_li_*) and K-Kv1.1 (*I_ci_*) corrected for the background signal were calculated for each *i*-cell. Ratio *R_i_* _=_ *I_li_*/*I_ci_* was calculated and averaged over 15–20 cells to obtain the *R_av_* value and the standard deviation.

To estimate the dissociation constant (*K_d_*) of A-HgTx complexes with K-Kv1.1, the dependence of *R_av_* on the added A-HgTx concentration (*L*) was fitted using the following equation:

*R_av_*(*L*)/*R_m_* = *L*/(*K_d_* + *L*) (2)

where *R_m_* is the maximal *R_av_* value corresponding to the saturation of binding. *K_d_* was estimated in independent measurements, averaged, and presented as mean ± SEM (*n* = 3).

To analyze the competitive binding of the studied peptide pore blocker and A-HgTx to K-Kv1.1 on the cellular membrane, *R_av_* data were plotted depending on the blocker concentration (*C*) at the constant A-HgTx concentration (*L*) and fitted with the following equation:

*R_av_*(*C*) = *R_av_*_0_/(1 + *C*/*DC*_50_) (3)

where *DC*_50_ is the blocker concentration displacing 50% of A-HgTx from the complex with K-Kv1.1, and *R_av_*_0_ is *R_av_* at *C* = 0.

The apparent dissociation constant (*K_ap_*) of the complex between the studied blocker and the K-Kv1.1 channel was calculated using the Cheng–Prusoff equation:

*K_ap_* = *DC*_50_/(1 + *L*/*K_d_*). (4)

Two independent experiments were performed, and the *K_av_* data were averaged and presented as mean ± SEM. Fifteen to twenty-five treated cell images were the average value for each point on the curves of A-HgTx binding to the K-Kv1.1 channel.

## 3. Results and Discussion

### 3.1. Features of K-Kv1.1wt Expression in Cells

**Figure 1:**
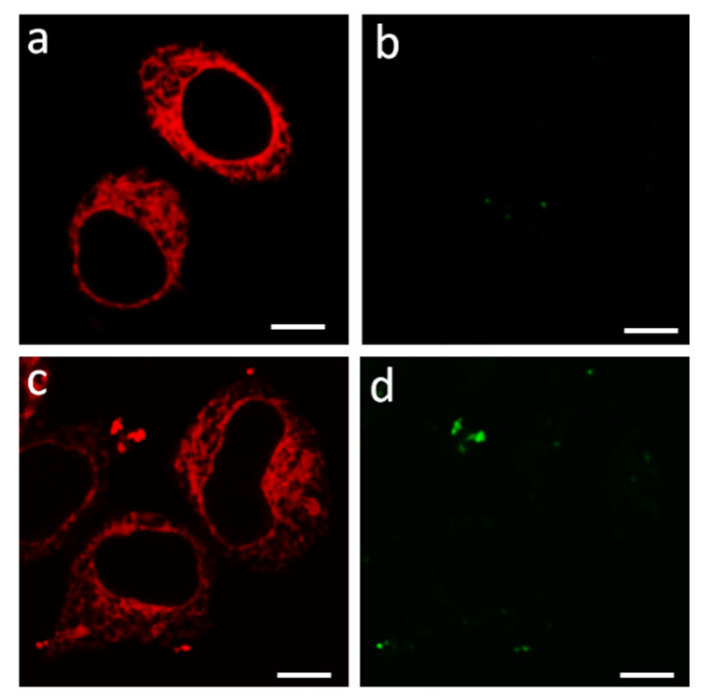
Fluorescent confocal images of Neuro2a cells expressing K-Kv1.1wt. (**a**,**c**) Typical distribution of K-Kv1.1wt in cells in the absence (**a**) or after incubation with 2 nM A-HgTx (**c**). (**b**,**d**) Distribution of fluorescence in the 500–530 nm range (the range of A-HgTx fluorescence) in the absence (**b**) or after incubation with 2 nM A-HgTx (**d**). Bar is 5 µm. The absence of K-Kv1.1wt expression in the plasma membrane is consistent with the absence of A-HgTx binding at the cell membrane.

**Figure 2:**
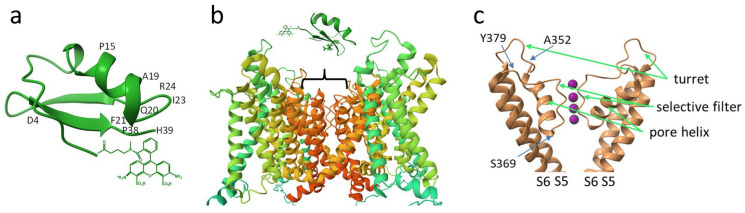
Schematic structures of A-HgTx and its complex with the Kv1.1 channel. (**a**) Structure of HgTx1 (pdb: 1HLY) labeled with Atto488 at the N-terminus. Positions of amino acid residues that are discussed in the text are indicated. (**b**) Characteristic structure of Kv1 channels in the membrane region (based on pdb: 2R9R). The position of the binding site of A-HgTx and other peptide blockers is shown by a bracket. (**c**) Structure elements of the outer pore region of the pore domain of the Kv1 channel (based on pdb: 2R9R), where the binding site of peptide pore blockers is situated. Front and back α-subunits are removed for clarity. Pink balls are potassium ions in the selectivity filter. S5 and S6 are transmembrane helices forming the pore domain. Positions of amino acid residues that are discussed in the text are indicated.

Transient transfection of Neuro2a cells with the pmKate2-KCNA1 plasmid leads to the appearance of a considerable portion of cells expressing K-Kv1.1wt. All these cells demonstrate a similar web-like distribution of the channels in the cytoplasm with a tendency to a higher concentration of them near the nucleus (Figure 1a). No explicit localization of K-Kv1.1wt is observed in the plasma membrane of these cells. The addition of a fluorescently labeled peptide pore blocker A-HgTx (Figure 2a) to cells does not lead to staining of the plasma membrane of living cells expressing or not expressing K-Kv1.1wt (Figure 1c,d). Since, as will be shown below, A-HgTx is a high-affinity ligand of Kv1.1 channels, it can be concluded that K-Kv1.1wt is not embedded in the plasma membrane.

This result is consistent with the data that native and heterologously expressed Kv1.1 channels are predominantly located in ER, where properly folded tetramers are formed and retained during biogenesis [9,28,29].

For the purposes of our study, membrane presentation of Kv1.1 channels has to be achieved. Accordingly, published data on residues determining the retention of the Kv1.1 channel in ER were analyzed in order to select mutations, which are implicated in the surface targeting of the Kv1.1 channel, but do not disrupt channel interactions with peptide pore blockers. Single-mutation A352P in the flexible “turret” of the outer pore region of the Kv1.1 channel was shown to enhance its surface expression, though a significant ER pool of mutant channels was still preserved [28]. Studies of the trafficking code for the Kv1.1 channel using mutations in the deep pore region revealed that the representation of the channel in the plasma membrane can be improved with the S369T substitution [29] and additionally enhanced with double mutation S369T/Y379K [30].

The scorpion toxin binding site is located at the extracellular side of the Kv1 channel pore (Figure 2b) and is formed by the residues of the P-loop, which connects the S5 and S6 transmembrane helices of the channel (Figure 2c) [31]. More precisely, the binding site comprises a “turret”, the most variable part of the P-loop, a short pore helix, a conservative potassium ion selectivity filter, and a short stretch of residues between the selectivity filter and the S6 transmembrane helix (Figure 2c). We assumed that S369, located deep in the pore region adjacent to the selectivity filter, most likely does not participate in the interaction with scorpion toxins, while the involvement of residues Y379 and A352 in the binding of peptide pore blockers is more likely (Figure 2c). In particular, the essential role of residue 379 in the binding of peptide toxins has been demonstrated [32]. Consequently, we have constructed the K-Kv1.1 channel with the S369T mutation and studied its properties.

### 3.2. Expression and Properties of K-Kv1.1 in Cells

**Figure 3:**
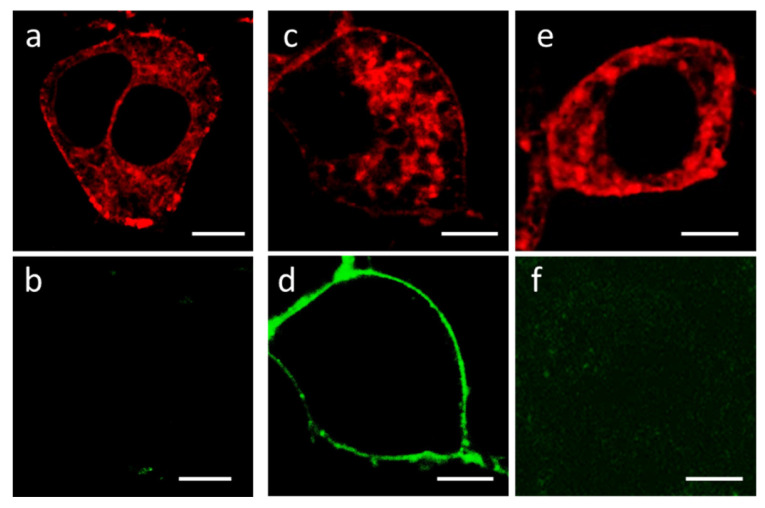
Fluorescent confocal images of Neuro2a cells expressing K-Kv1.1. (**a**,**c**,**e**) Typical distribution of K-Kv1.1 in cells in the absence (**a**) or after the addition of 2 nM A-HgTx (**c**), or in the presence of 2 nM A-HgTx and 10 nM HgTx1 (**e**). (**b**,**d**,**f**) Distribution of fluorescence in the 500–530 nm range (the range of A-HgTx fluorescence) in the absence (**b**) or after the addition of 2 nM A-HgTx (**d**), or in the presence of 2 nM A-HgTx and 10 nM HgTx1 (**f**). Bar is 5 µm. The expression of K-Kv1.1 in the plasma membrane ensures the binding of A-HgTx at the cell membrane (**d**) and the competitive displacement of A-HgTx from complexes with K-Kv1.1 by an excess of non-fluorescent HgTx1 (**f**).

In contrast to the K-Kv1.1wt channel (Figure 1), the mutated K-Kv1.1 channel expressed in Neuro2a cells demonstrates distinct staining of the plasma membrane in addition to cytoplasmic distribution (Figure S1a,c,e and Figure 3a,c,e). Plasma membrane staining is also observed when the K-Kv1.1 channel is expressed in Hek293 cells (Figure S2a). Membrane presentation of K-Kv1.1 was confirmed by the binding of A-HgTx (Figure 3, Figures S1 and S2). The membrane presentation of mutated Kv1.1 does not depend on the origin of the fluorescent protein, as demonstrated by the replacement of mKate2 with a cyan fluorescent protein (Figure S3). A-HgTx can be displaced from complexes with K-Kv1.1 on the membrane using an excess of non-labeled HgTx1 (Figure 3 and Figure S1). This indicates that the sites of binding of A-HgTx and HgTx1 coincide or overlap considerably.

**Figure 4:**
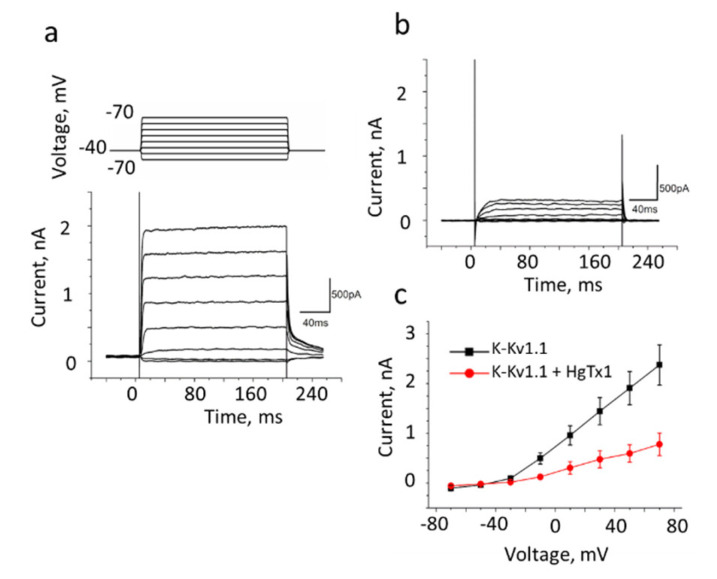
The whole-cell recording of currents in Neuro2a cells expressing K-Kv1.1 without (**a**) or with added 2 nM HgTx1 (**b**). The membrane potential was changed from −70 to +70 mV in increments of 20 mV using 200 ms pulses (as shown in the insert at the top of panel a) that were applied at 20 s intervals. The holding potential (−40 mV) was maintained before and after depolarizing pulses. Representative series of currents are shown. (**c**) Dependence of the transmembrane current (mean± SEM, *n* = 10) on the applied potential in the absence (black color) or after addition (red color) of 2 nM HgTx1.

The conductivity of the K-Kv1.1 channel in Neuro2a cells was characterized by the patch-clamp technique in the whole-cell configuration. Potassium currents with rapid activation and slow inactivation were detected (Figure 4a), similar to the typical currents of rodent and human Kv1.1 channels [33,34,35]. The measured dependence of the current on the applied voltage revealed a positive current threshold in cells expressing K-Kv1.1 at a potential above −30 mV and an increase in the current amplitude that is proportional to the voltage in the range from −30 to 70 mV (Figure 4a,c). The 2–4 nA outward current was detected at 70 mV in cells expressing K-Kv1.1 (Figure 4a). The application of HgTx1 (2 nM), the high-affinity peptide blocker of the Kv1.1 channel [36], caused a decrease in the peak current amplitude by 73 ± 8% (*n* = 10) at an applied voltage varying from −10 to 70 mV (Figure 4b,c). In non-transfected Neuro2a cells, outward voltage-dependent ion currents are low (0.1–0.2 nA) to interfere noticeably with the K-Kv1.1-related currents in the transfected cells (Figure S4 and published data [23]). These “background” currents are not related to Kv1 channels because they are insensitive to the application of Kv1-channel blockers ChTx [23] and HgTx1 (Figure S4). At least in part, the “background” currents are responsible for residual currents after the addition of HgTx1 to cells expressing K-Kv1.1 (Figure 4b,c).

Based on electrophysiology data, one can conclude that K-Kv1.1 expressed in Neuro2a cells is a functionally active voltage-gated channel. K-Kv1.1 preserved the binding site of scorpion toxins, and specific peptide pore blockers, such as HgTx1, block ion current through the channel.

**Figure 5:**
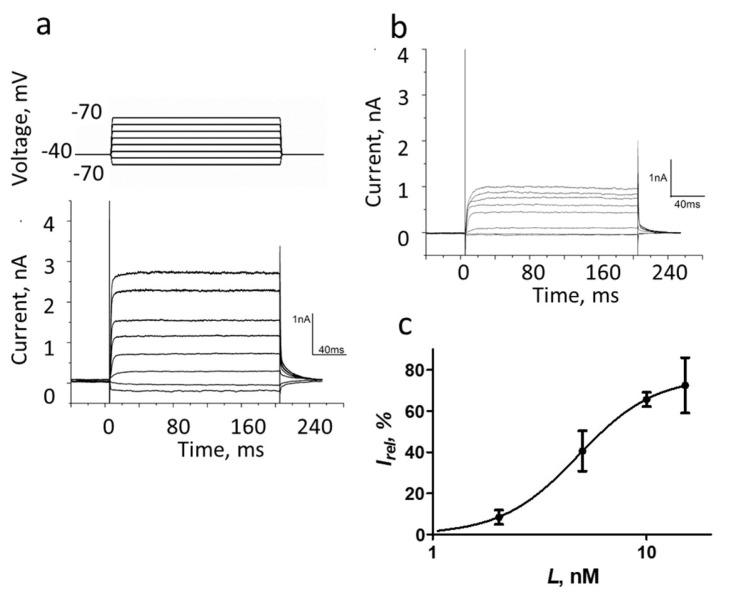
The whole-cell recording of currents in Neuro2a cells expressing K-Kv1.1 in the absence (**a**) or after the addition of 5 nM A-HgTx (**b**). The membrane potential was changed from −70 to +70 mV in increments of 20 mV using 200 ms pulses (as shown in the insert at the top of panel (**a**)) that were applied at 20 s intervals. Finally, the potential returned to the holding potential (−40 mV). Representative series of currents are shown. (**c**) Dependence of inhibition of K-Kv1.1 current on the concentration of A-HgTx at +50 mV. Data are mean ± SEM (*n* = 6).

The ability of A-HgTx to block ion currents through the K-Kv1.1 channel was verified with the patch-clamp technique. The 5 nM A-HgTx induced approximately a 50% decrease in the amplitude of the current through the K-Kv1.1 channel at an applied voltage varying from −30 to 70 mV (compare Figure 5a,b). The inhibition of the current depended on the concentration of added A-HgTx. At the +50 mV voltage, the maximum (ca. 75%) current inhibition was achieved at the concentration of A-HgTx above 15 nM, while the concentration of the A-HgTx-inhibiting current by 50% (*IC*_50_) was 4.8 ± 0.2 nM (Figure 5c). Thus, fluorescently labeled HgTx1 possesses a nanomolar affinity for the Kv1.1 channel and the ability to block the channel pore.

### 3.3. Localization of K-Kv1.1 in Cells

While the plasma membrane localization of K-Kv1.1 was confirmed due to the binding of A-HgTx (Figure 3), the intracellular localization of K-Kv1.1 in Neuro2a cells was characterized by laser scanning confocal microscopy using fluorescent markers of cellular organelles.

**Figure 6:**
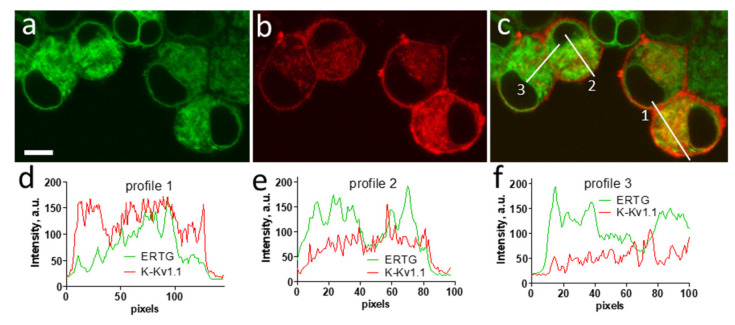
Confocal images showing distributions of K-Kv1.1 (**a**) and ER marker ERTG (**b**) in living Neuro2a cells. (**c**) Merged image. Yellow color shows the co-localization of K-Kv1.1 and ERTG in ER. Scale bar is 10 µm. (**d**–**f**) Profiles of ERTG and K-Kv1.1 fluorescence distribution along white lines marked in panel (**c**).

**Figure 7:**
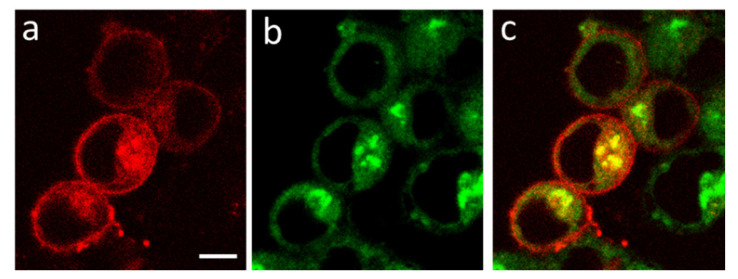
Confocal images showing distributions of K-Kv1.1 (**a**) and Golgi apparatus marker NCer (**b**) in living Neuro2a cells. (**c**) Merged image. Yellow color shows the co-localization of K-Kv1.1 and NCer in the trans-Golgi apparatus. Scale bar is 10 µm.

Intracellular distributions of K-Kv1.1 and the endoplasmic reticulum marker ERTG have similar patterns (Figure 6a–c), though fine features of their localization do not correlate in many cases (Figure 6d–f). The analysis performed using the marker of the trans-Golgi apparatus NCer shows that a noticeable fraction of K-Kv1.1 definitely localizes in the Golgi apparatus (Figure 7). Thus, only a part of K-Kv1.1 assembled in ER is finally transported to the plasma membrane, while its considerable fraction is retained in the compartments involved in the channel traffic, in particular, in the Golgi apparatus and ER. Two days after transfection, the expression of K-Kv1.1 in the plasma membrane becomes more pronounced than 24 h after transfection, indicating rather slow channel traffic to the plasma membrane. No evidence of the traffic and accumulation of K-Kv1.1 in mitochondria was observed in our study (Figure S5).

The K-Kv1.1 channel, expressed in the plasma membrane, can probably undergo endocytosis with further accumulation in lysosomes. This conclusion is based on the partial co-localization of K-Kv1.1 with the endosomal marker TR488 (Figure S6) and the lysosomal marker LTG (Figure S7). Endosomes and lysosomes may participate in the disposal or recycling of channels.

### 3.4. Interactions of Peptide Pore Blockers with K-Kv1.1

**Figure 8:**
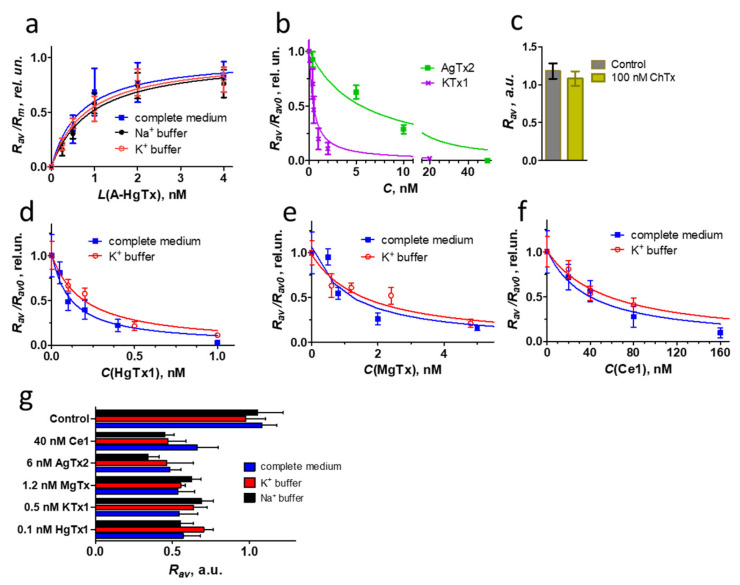
Quantitative analysis of K-Kv1.1 interactions with different peptide pore blockers. (**a**) Concentration-dependent binding of A-HgTx to K-Kv1.1, which was measured as the dependence of *R_av_* on the concentration *L* of A-HgTx added to Neuro2a cells and normalized to the maximum *R_av_* value (*R_m_*). Measurements were performed in a complete medium or a buffer with either 150 mM KCl (K^+^ buffer) or 4 mM KCl and 150 mM NaCl (Na^+^ buffer). The complete medium and Na^+^ buffer maintains the closed state of the channels, while the K^+^ buffer favors the open state of the channels. (**b**,**d**–**f**) Competitive binding of A-HgTx (2 nM) and different peptide pore blockers to K-Kv1.1. *C*—concentration of non-labeled peptide pore blocker. Representative dependencies are shown. Measurements were performed in a complete medium (**b**,**d**–**f**) or in a K^+^ buffer that favored the open state of the channels (**d**–**f**). (**c**) ChTx (100 nM) is not able to compete with A-HgTx (2 nM) for binding to K-Kv1.1. Control—binding of A-HgTx (2 nM) to K-Kv1.1 in the absence of ChTx. (**g**) Competitive binding of A-HgTx (2 nM) and different peptide pore blockers (concentrations are indicated in the diagram) to K-Kv1.1 in a complete medium, K^+^ and Na^+^ buffers. Concentrations of peptide pore blockers were selected to displace 45–60% of A-HgTx from the complexes with K-Kv1.1 in a complete medium.

A-HgTx binding to K-Kv1.1 on the plasma membrane is concentration-dependent (Figure 8a). It is detected at the ligand concentration higher than 0.5 nM and saturated at ca. 2 nM. Analysis of A-HgTx binding to K-Kv1.1 with Equation (2) shows that *K_d_* of the complex is 0.7 ± 0.2 nM. Taking into account the high affinity of A-HgTx for Kv1.1 and the absence of non-specific binding to the cell membrane, this ligand is applicable for fluorescent imaging of Kv1.1 channels in cells. In accordance with the binding profile of HgTx1 [36], A-HgTx is not a selective ligand of the Kv1.1 channel. It interacts with the Kv1.3 channel also (*K_d_* = 0.30 ± 0.13 nM) [23].

In addition, A-HgTx is intrinsically suitable for use as a component of an analytical cellular system when studying the interactions of natural or synthetic pore blockers with the Kv1.1 channel. Such a system can be based on the analysis of competition between fluorescent and tested ligands for binding to a channel on the cell membrane [23]. Confirming this supposition, the known peptide blockers of the Kv1.1 channel KTx1, MgTx, HgTx1, and AgTx2 compete for the K-Kv1.1 binding with A-HgTx in a concentration-dependent manner (Figure 8b,d–f). Reasonably, ChTx, which has a very low affinity for Kv1.1 [37], does not affect the binding of A-HgTx to K-Kv1.1 at a concentration as high as 100 nM (Figure 8c). The analysis of competitive binding curves (Figure 8b,d–f) using Equations (3) and (4) allows one to calculate the *K_ap_* of complexes formed by the studied peptide blockers with K-Kv1.1 in a complete medium (Table 2). The *K_ap_* value of HgTx1 (Table 2) confirms the peptide affinity estimated previously by an approach based on the Rb^+^ efflux through Kv1.1 channels in mammalian cells [36]. For AgTx2, the *K_ap_* value (Table 2) is close to one of two reported concentrations of the peptide that inhibits current through Kv1.1 channels in oocytes by 50% [37,38]. The *K_ap_* value of MgTx (Table 2) is similar to the affinity revealed by the Rb^+^ efflux approach [36] but deviates from the value estimated by the patch-clamp technique [18]. The measured KTx1 affinity (Table 2) is tenfold better than its affinity determined by the voltage clamp technique on oocytes [39]. It should be noted that deviations of peptide activities measured by different techniques are a typical situation in the literature. The reasons for these deviations require special investigation and may include the origin of the channels used for measurements (murine, rat, human), errors in determining the peptide concentration (especially when small amounts of peptides have been isolated from natural sources), differences in buffer compositions (ionic strength strongly affects the peptide–channel interactions), the state of the channel (open vs. close) and some others. The conclusion, which can be drawn based on the measurements performed (Figure 8, Table 2), is that the analytical system based on A-HgTx ligand and cell expressing K-Kv1.1 channels is applicable for studies of peptide pore blockers of Kv1.1, including the determination of *K_ap_* of the complexes formed by these peptides.

Exploring the potential of this analytical system, we have measured the *K_ap_* of the complex between the recombinant peptide Ce1 and the Kv1.1 channel (Figure 8c, Table 2) and classified Ce1 as a moderately active ligand of this channel. Toxin Ce1 was found in the venom of the scorpion *Centruroides elegans* and characterized as a high-affinity pore blocker of the Kv1.3 channel (*IC*_50_ = 0.7 nM) [26]. Its activity for the Kv1.1 channel was not previously reported.

Ce1 is a member of the α-KTx2 subfamily of scorpion toxins and is a close homolog of the well-studied toxins HgTx1, MgTx, and noxiustoxin (NTx, Table 3). NTx does not block the Kv1.1 channel at concentrations up to 25 nM [33]. Thus, Ce1 surpasses the closest homolog NTx in affinity for Kv1.1, but inferiors MgTx and especially HgTx in this characteristic (Table 2).

A simple comparison of the sequences and activities of HgTx1, MgTx, Ce1, and NTx shows that the variable residues, which can potentially determine variations in the peptide binding to the Kv1.1 channel, are residues 4, 15, 19, 20, 21, 23, 24, 38, and 39 (Table 3, Figure 2a). Molecular modeling data confirm that H39 is among the main determinants of the binding of HgTx1 and MgTx to Kv1.1–Kv1.3 channels [40]. Besides the replacement of H39N, the decreased affinity of NTx for the Kv1.1 channel compared to MgTx and HgTx1 is explained by the P15K substitution since two proline residues in the middle of the α-helix contribute to the bending of the helix, which is important for interaction with the bulky Y381 residue of the Kv1.1 channel. This explanation of the decreased affinity for Kv1.1 can be translated to Ce1, which also contains H39N and P15K substitutions (Table 3). The roles of other variable residues still need to be clarified.

### 3.5. Open vs. Closed K-Kv1.1 Channels in Interactions with Peptide Pore Blockers

Traditional techniques, such as electrophysiological methods and the Rb^+^ efflux approach, analyze the interactions of peptide pore blockers with ion channels in the open state. In contrast, the analytical system described in our paper operates when most channels are in a closed state. At the same time, this system provides a rare possibility to compare the interactions of peptide pore blockers with the closed and open forms of the Kv1.1 channel by replacing sodium ions in the outer medium with potassium ions, which are known to induce depolarization of the plasma membrane [41] and the opening of the Kv1.1 channels. Assuming that various physiological factors can influence the gating kinetics, as well as the duration of the open or closed state of the channel, the study of the state-dependent inhibition of the Kv1.1 channel is of therapeutic importance.

In our experiments, plasma membrane depolarization was induced by replacing a complete medium or buffer containing 150 mM NaCl with a buffer comprising 150 mM KCl. Assuming that the intracellular concentration of K^+^ ions is 140 mM [42], the resting potential of the membrane at the 150 mM extracellular concentration of K^+^ ions, calculated by the Nernst equation, is slightly positive (+2 mV). K-Kv1.1 channels are open at this potential (Figure 4a). Analysis of the concentration dependences of A-HgTx interaction with K-Kv1.1 in the Na^+^ and K^+^ buffers favoring either the closed or open state of the channels did not reveal significant changes in the calculated *K_d_* values, 0.9 ± 0.3 and 0.8 ± 0.1 nM, respectively (Figure 8a). Moreover, a comparison with the *K_d_* value measured for A-HgTx in a complete medium shows that the components of the cell growth medium and fetal calf serum do not interfere with the peptide-channel interactions.

The analysis was extended from labeled to non-labeled peptide pore blockers, and the interactions of HgTx1, MgTx, KTx1, AgTx2, ChTx, and Ce1 with open and closed K-Kv1.1 were compared (Figure 8g). Since the interaction of A-HgTx with the K-Kv1.1 channel does not depend on the channel state, variations in the competition of non-labeled ligands with A-HgTx for binding to open and closed K-Kv1.1 can be used as an indicator of changes in the affinity of these ligands for the channel in different states. For the analysis, the concentrations of peptides were selected, which displace 45–60% A-HgTx (2 nM) from complexes with closed K-Kv1.1 channels in a complete medium. At such concentrations, the assay is very sensitive to any changes in the peptide–channel interactions. Comparison of the A-HgTx displacement from complexes with K-Kv1.1 by the studied peptides in the complete medium as well as in Na^+^ and K^+^ buffers did not reveal significant changes for any of the peptides (Figure 8g). This means that the channel state (closed or open) does not affect the affinity of K-Kv1.1 for HgTx1, MgTx, KTx1, AgTx2, ChTx, or Ce1, and the components of the cell growth medium and fetal calf serum do not interfere with the peptide–channel interactions. To further confirm this conclusion, detailed titration experiments were performed for three selected peptides (HgTx1, MgTx, and Ce1) in the K^+^ buffer (Figure 8d–f). *K_ap_* values determined for peptide complexes with open channels (0.05 ± 0.01, 0.5 ± 0.2, and 18 ± 4 nM for HgTx1, MgTx, and Ce1, respectively) were found to be insignificantly different from the corresponding *K_ap_* values obtained for closed channels (Table 2).

In the absence of voltage-dependent activation of the channel, peptide toxins, which bind the channel from its external side, block the channel in its resting closed state. This state is characterized by a conductive conformation of the selectivity filter and a closed activation gate of the channel, which is formed by the bundle-crossing of S6 transmembrane helices of four α-subunits [43]. The binding of the peptide blocker is accompanied by occlusion of the channel pore by the lysine side chain and the formation of multiple bonds with the residues of the P-loop connecting the transmembrane helices S5 and S6 of the channel (Figure 2b,c) [40,44,45]. Voltage-dependent activation leads to structural transitions in the channel, starting with the movement of S4 helices, which sense the transmembrane potential, followed by alterations in the conformation of the S6 helices and the opening of the inner activation gate. Since the high-resolution structures of the Kv1.1 channel in both closed and open states have not yet been solved, it is unclear whether these conformational changes are translated into the P-loop region. The results of our studies obtained for several peptide blockers, which are characterized by different sets of peptide–channel interactions, allow us to conclude that the conformation of the P-loop (including the selectivity filter region) of the Kv1.1 channel (Figure 2c) is not essentially changed during the transition from the closed to the open state. This experimental result confirms the data of molecular dynamics simulations on the structural stability of the selectivity filter in the open state of the Kv1.1 channel [46], which preserves effective interaction of the pore-occluding Lys residue of the peptide blocker. At the same time, one cannot exclude that the peptide binding may lead to the stabilization of the conductive conformation of the Kv1 channel, as shown for the charybdotoxin–Kv1 channel complex [47].

## 4. Conclusions

The results of our studies demonstrate that A-HgTx possesses a high (subnanomolar) affinity for the Kv1.1 channel and can be used for fluorescent imaging of this channel in mammalian cells.

The human Kv1.1 channel is retained in the cytoplasm, but in accordance with the previous data [29], the S369T mutation strongly enhances the embedding of the channel into the plasma membrane. At the same time, a considerable portion of the mutated channel is still retained in the Golgi apparatus and ER. The S369T-mutated fluorescent Kv1.1 channel preserves functional activity and the binding site of peptide pore blockers.

The combination of the A-HgTx ligand, mammalian cells expressing the fluorescent mutated Kv1.1 channel, and confocal microscopy forms the basis for an analytical system and an associated technique that enable the study of interactions of non-fluorescent peptide pore blockers with this channel and characterization of the affinities of peptide-channel interactions. An analogous system based on the Kv1.3 channel was reported by us earlier [23].

Using a new analytical system, it was demonstrated that Ce1 peptide from the venom of the scorpion *Centruroides elegans* is a pore blocker with nanomolar affinity for the Kv1.1 channel, and the affinities of HgTx1, MgTx, KTx1, AgTx2, ChTx, and Ce1 for the Kv1.1 channel do not change after the channel transition from the closed to the open state. In the absence of high-resolution structural data on Kv1.1, the results obtained indicate that structural rearrangements occurring during the channel transition to the open state hardly alter the conformation of the P-loop (including the selectivity filter) involved in the formation of the binding site of peptide pore blockers.

The technique developed by us expands the list of available methods for studying ion channels and their ligands. Starting with the Kv1.3 channel [23], we, as shown above, have successfully extended the application of this technique to the Kv1.1 channel and are working on its further extension to other types of channels. Investigations of ion channels and their ligands with the new technique may include the search for Kv1-specific blockers in natural venoms, comparative analysis of mutated or newly designed peptides in a drug discovery research, the study of ligand–channel interaction interface, the search for and design of state-dependent channel blockers with altered affinity for open and closed channels as well as the development of fluorescent ligands of Kv1 channels.

## Figures and Tables

**Table 1:** Oligonucleotide primers.

Notation	Nucleotide Sequence *
Kcna1-f1	5′-TTCTCAGATCT**ATG**ACGGTGATGTCTGGGGAGAACGT-3′
Kcna1-r1	5′-TTCTCAAGC**TT****A**AACATCGGTCAGTAGCTTGCTCTTA-3′
Kcna1m1-f1	5′-*GCGGTGGTG**ACC**ATGACCACTG* TAGGATACG-3
Kcna1m1-r1	5′-*CAGTGGTCAT**GGT**CACCACCGC* CCACCAGAA-3′
Fl-f1	5′-TTCTTCGCTAGCGCTACCGGTCGCCACC-3′
Cfp-r1	5′-TCTAGATCTGAGTCCAGACCCTCCGCCACCGCGGTACAGCTCGTCC
Ce1-f1	5′-TCTCGGTACCGAAAACCTGTATTTTCAGACCGTGATCAACGTGAAATGCACC-3′
Ce1-f2	5′-AAACCGTGCAAAGATCTGTATGGTCCGCATGCAGGTGCGAAATGCATGAAC-3′
Ce1-r1	5′-AGATCTTTGCACGGTTTCAGACACTGTTTCGGGCTGGTGCATTTCACGTTG-3′
Ce1-r2	5′-TCTCAAGCTTAGTTATTATAGCATTTACATTTACCGTTCATGCATTTCGCA

* Sites of restriction enzymes are underlined. Start and stop codons (ATG and TTA) in forward Kcna1-f1 and reverse Kcna1-r1 primers, as well as codons for T369, ACC and GGT, in forward Kcna1m1-f1 and reverse Kcna1m1-r1 primers are marked in bold. In the Cfp-r1 reverse primer, the nucleotide sequence complementary to the 3′-terminal sequence of a *TagCFP* gene is underlined. Overlapping sequences are shown in italics.

**Table 2:** The *K_ap_* values for complexes formed by K-Kv1.1 with the studied peptide pore blockers and published data on the affinities of these blockers.

	HgTx1	KTx1	AgTx2	MgTx	Ce1
*K_ap_*, nM	0.03 ± 0.02	0.12 ± 0.08	1.5 ± 0.7	0.3 ± 0.2	11 ± 5
*IC*_50_ *, nM	0.031 ^a^	1.1 ^b^	0.044 ^c^, 2 ^d^	0.144 ^a^, 4.2 ^e^	- ^f^

* Concentration of peptide inhibiting K^+^ or Rb^+^ current through the channel by 50%; ^a^ Rb^+^ efflux from HEK293 cells [36]; ^b–d^ rat channels, oocytes, two-electrode voltage clamp [39] ^b^, [37] ^c^, [38] ^d^; ^e^ human channels, tsA201 cells, patch-clamp [18]; ^f^ not available.

**Table 3:** Alignment of sequences and homology of HgTx1, MgTx, Ce1, and NTx.

Toxin	Amino Acid Sequence	Homology, %			
		HgTx1	MgTx	Ce1	NTx
HgTx1 *	1 10 20 30 T**V**I**D**VKCTSPKQC**LP**PCK**AQF**G**IR**AGAKCMNGKCKCY**PH**	100	90	77	70
MgTx	T**I**I**N**VKCTSPKQC**LP**PCK**AQF**G**QS**AGAKCMNGKCKCY**PH**	90	100	77	80
Ce1	T**V**I**N**VKCTSPKQC**LK**PCK**DLY**G**PH**AGAKCMNGKCKCY**NN**	77	77	100	87
NTx	T**I**I**N**VKCTSPKQC**SK**PCK**ELY**G**SS**AGAKCMNGKCKCY**NN**	70	80	87	100

* Variable residues are shown in bold.

## Data Availability

Not applicable.

## Supplementary Materials

The following supporting information can be downloaded at: https://www.mdpi.com/article/10.3390/membranes13070645/s1, Figure S1: Overview fluorescent confocal images of Neuro2a cells expressing K-Kv1.1; Figure S2: Overview fluorescent confocal images of Hek293 cells expressing K-Kv1.1, which were stained with 2 nM A-HgTx; Figure S3: Confocal images showing distribution of C-Kv1.1 (a) and A-HgTx (2 nM) (b) in Neuro 2a cells; Figure S4: The whole-cell recording of currents in non-transfected Neuro2a cells without (a) or with the added 2 nM HgTx1 (b); Figure S5: Confocal images showing distribution of K-Kv1.1 (a) and the mitochondrion probe R123 (b) in Neuro2a cells; Figure S6: Confocal images showing distribution of K-Kv1.1 (a) and the endosome probe TR488 (b) in Neuro2a cells; Figure S7: Confocal images showing distribution of K-Kv1.1 (a) and the lysosome probe LTG (b) in Neuro2a cells.
