# Supplementary material for: Interactions of the Kv1.1 Channel with Peptide Pore Blockers: A Fluorescent Analysis on Mammalian Cells
Source: Membranes (Basel). 2023 Jul 4;13(7):645. doi: 10.3390/membranes13070645 (PMC10383195; doi:10.3390/membranes13070645)
Supplement: Supplementary file 1 [file membranes-13-00645-s001.zip › membranes-2466919-supplementary.pdf]

Supplementary materials

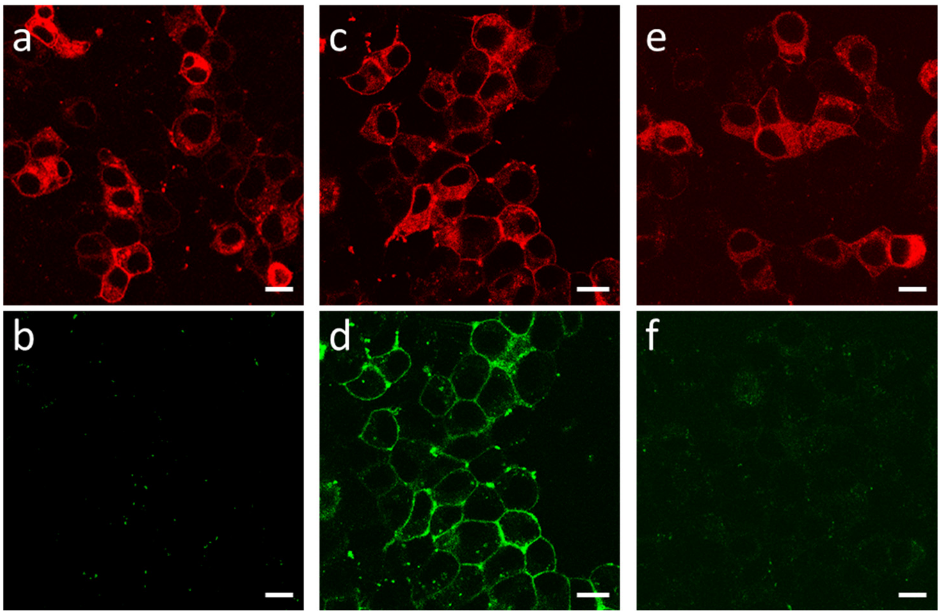

Figure S1. Overview fluorescent confocal images of Neuro2a cells expressing K-Kv1.1. (a,c,e) Typical distribution of K-Kv1.1 in cells in the absence (a) or in the presence of 2 nM A-HgTx (c), or in the presence of 2 nM A-HgTx and 10 nM HgTx (e). (b, d, f) Distribution of fluorescence in the 500-530 nm range (the range of A-HgTx fluorescence) in the absence (b) or in the presence of 2 nM A-HgTx (the A-HgTx binding to K-Kv1.1 at the cellular membrane) (d), or in the presence of 2 nM A-HgTx and 10 nM HgTx (the concurrent displacement of A-HgTx from the complexes with K-Kv1.1 by an excess of non-fluorescent HgTx1) (f). Bar is 20 μm.

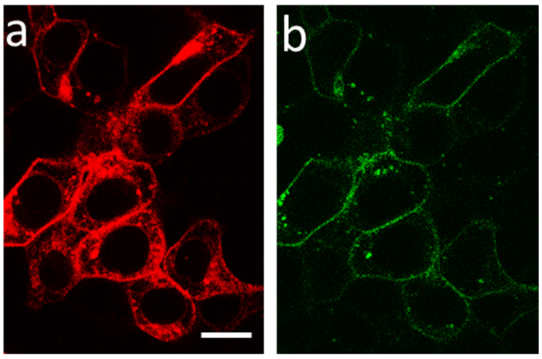

Figure S2. Overview fluorescent confocal images of Hek293 cells expressing K-Kv1.1, which were stained with 2 nM A-HgTx. Typical distributions of K-Kv1.1 (a) and A-HgTx (b) are shown. Bar is 20 μm.

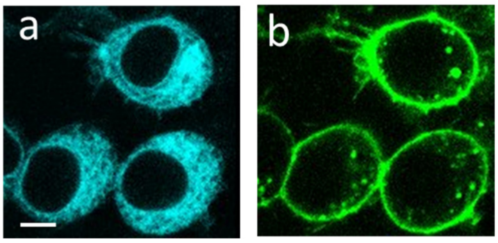

Figure S3. Confocal images showing distribution of C-Kv1.1 (a) and A-HgTx (2 nM) (b) in Neuro 2a cells. Scale bar is 10 μm.

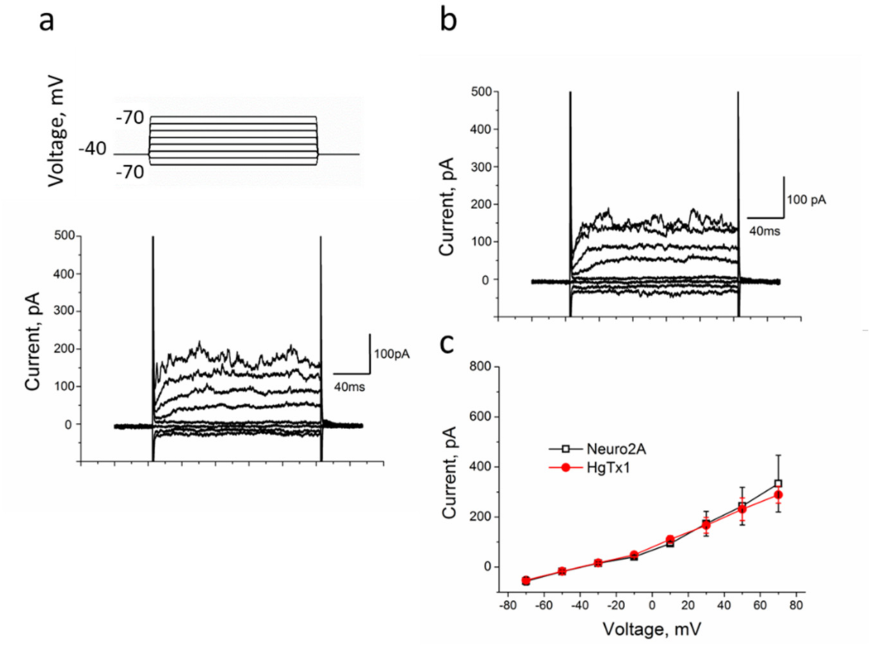

Figure S4. The whole-cell recording of currents in non-transfected Neuro2a cells without (a) or with added 2 nM HgTx1 (b). The membrane potential was changed from –70 to +70 mV in increments of 20 mV using 200 ms pulses (as shown in the insert at the top of panel a) that were applied at 20 s intervals. The holding potential (–40 mV) was maintained before and after depolarizing pulses. Representative series of currents are shown. (c) Dependence of the transmembrane current (mean± SEM, n=10) on the applied potential in the absence (black color) or after addition (red color) of 2 nM HgTx1.

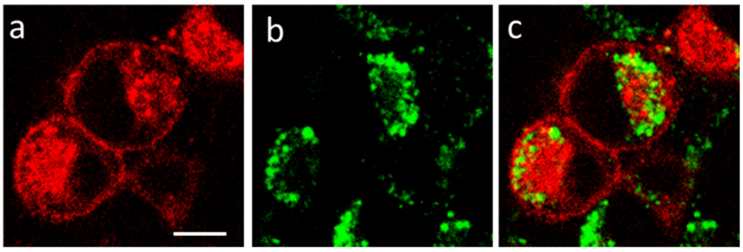

Figure S5. Confocal images showing distributions of K-Kv1.1 (a) and the mitochondrion probe R123 (b) in Neuro2a cells. (c) Merged image of K-Kv1.1 and R123 fluorescence, where the absence of yellow indicates the absence of co-localization of K-Kv1.1 and R123 in mitochondria. Scale bar is 10 µm.

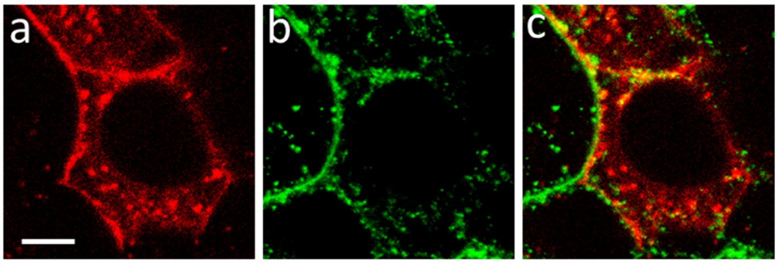

Figure S6. Confocal images showing distribution of K-Kv1.1 (a) and the endosome probe TR488 (b) in Neuro2a cells. (c) Merged image of K-Kv1.1 and TR488 fluorescence, where yellow color indicates the co-localization of K-Kv1.1 and TR488 in endosomes. Scale bar is 5 µm.

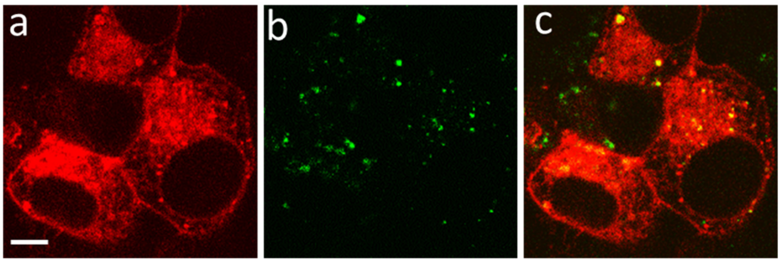

Figure S7. Confocal images showing distribution of K-Kv1.1 (a) and the lysosome probe LTG (b) in Neuro2a cells. (c) Merged image of K-Kv1.1 and LTG fluorescence, where yellow color indicates the co-localization of K-Kv1.1 and LTG in lysosomes. Scale bar is 5 μm.
